# Is Perceived Autonomy Support Provided by a Coach Related to the Intention of Injury Preventative Behavior Among National and International Level Golfers?

**DOI:** 10.3389/fspor.2021.569590

**Published:** 2021-03-09

**Authors:** James Parker, Urban Johnson, Andreas Ivarsson

**Affiliations:** Center of Research on Welfare, Health and Sport, Halmstad University, Halmstad, Sweden

**Keywords:** autonomy support, coaching, golf, injury prevention, network analysis

## Abstract

The successful implementation of injury prevention programs is reliant on athletes and coaches accepting, adopting, and complying with behaviors that reduce injury risk. Exploring factors, such as motivation and planned behavior, that might increase the frequency of these behaviors warrants investigation. The aim of the study was to investigate the complex interaction between perceived autonomy support, self-determined motivation, planned behavior, and how this relates to golfers self-reported intention injury preventative behavior. A total of 60 golfers completed questions on psychological measures of perceived autonomy support from coaches, autonomous motivation, and intentions of injury preventative behavior. A neural network model analysis was performed to investigate the strength of connection between covariates and construct a network structure. Analysis of results was performed by assessing edge strengths and node centrality to guide inference of the network topology. The most central node was autonomous regulation and the results showed one cluster comprising positive interactions between perceived autonomy support, effort of injury preventative behavior, and frequency of injury preventative behavior. When aiming to encourage injury preventative behavior, coaches should consider giving feedback that supports autonomous motivation since it is positively associated with effort and frequency of injury preventative behavior among high-level golfers. Injury prevention programs should include strategies to improve the athlete's autonomous motivation to carry out preventive activities.

## Background

Sports injuries are an inherent part of sports participation for many athletes. For high-level ahtletes the risk of injury may be higher due to the demands to repeatadly perform to, for example, secure an income. Professional golfer players are no exception to the demands to repeatedly perform to cover earnings and often seek pain relief treatment for overuse injuries in order to tolerate continued play (Smith and Hillman, 2012). The necessity for continued participation combined with the travel schedule makes continued medical support problematic for high-level golfers (Hawkes et al., 2013; O'Connor and Hawkes, 2013), consequently high-level golfers need to take responsibility for their own injury prevention strategies. Investigating how factors such as motivation and behavioral intensions influence the injury preventive strategies an athlete adopts can give practitioners insight that might help increase the frequency of injury preventive behaviors performed by their athletes. The goal of this study was to, through a complex systems approach, investigate potential interactions between perceived autonomy support and the intention of injury preventative behavior.

## Injury Preventative Behavior

Research over the past decades has provided a wide array of preventative strategies for a range of injuries in many sports. For instance, golf-related injuries can be reduced through strategies like muscular strengthening, pre-game warm-up, adjustment of individual technique, following rules and regulations, and using the correct equipment (Thériault and Lachance, 1998). The success of injury prevention strategies is reliant on athletes and coaches accepting, adopting, and complying with these to ultimately performing the specific behaviors required (Vriend et al., 2017). The performance of a specific behavior, such as a pre-game warm-up, can be considered as injury preventative strategies. Thus, an injury preventative behavior is when an athlete implements and carry out a specific preventative measure to reduce the risk of injury or re-injury.

## Motivation and Perceived Autonomous Support

Research has shown that adherence to injury-preventative behaviors is often poor (Verhagen et al., 2010; Chan et al., 2011) not seldom because of athlete's motivation (Andersson et al., 2019) and it is, therefore, important to understand factors that influence take-up and adherence to an injury prevention program. The self-determination theory (SDT; Deci and Ryan, 1985; Ryan and Deci, 2017) posits that motivational regulations for specific behaviors can be classified into three broad categories (i.e., autonomous, controlled, amotivation). When a behavior is self-initiated and coherent with one's deeply-rooted values, it is, according to the SDT, regulated by autonomous regulation (Deci and Ryan, 1985, 2002). Controlled regulation, which is juxtaposed to autonomous regulation, can also regulate behavior and the reasons are considered external to the individual and often perceived by the individual as feelings of being pressured or coerced into a behavior. Individuals that show high levels of autonomous regulation are more likely to show sustainable and adaptive behavior (Deci and Ryan, 2002). Research frequently shows that athletes with higher autonomous regulations show greater intention and adherence to injury preventative behaviors (Chan D. K. and Hagger, 2012).

The support of significant others can have an indirect effect on an athletes injury preventive behaviors via motivational regulations (see e.g., Hagger et al., 2015). For example, physiotherapists who provide autonomy supportive climates by establishing a relationship with an injured athlete can increase the players confidence and improve adherence to the rehabilitation program (Carson and Polman, 2017; Ardern et al., 2018). The perceived autonomy support provided by significant others, such as coaches may not have the same influence on motivation and injury preventative behaviors as the perceived autonomy support provided by sports medicine staff who are able to build trust with the athlete due, partly, to their in depth knowledge on sports injuries (Chan et al., 2011). These differences may be problematic for a sports coach looking to foster greater adherence to injury preventative behaviors but who lack in-depth knowledge on injuries. Chan D. K. and Hagger (2012) showed that coaches who create motivational environments that foster self-determined motivation can positively influence an athlete's injury preventative behaviors. This phenomenon is of interest within golf, given the travel schedules of high-level golfers and their lack of continuous support from sports medicine staff which places a high demand on the individual golfers' motivation and capacity to adopt and adhere to an injury prevention program.

## Theory of Planned Behavior

Constructs of SDT can be viewed as mechanisms that explain the manifestation of behaviors to satisfy basic psychological needs (autonomy, competence, and relatedness). The self-determination theory addresses the mediators in the relation between the manifestation of behaviors and the situation specific mediators that influence the enactment of a behavior that momentarily obtains the desired outcome (Prentice et al., 2019). The theory of planned behavior (TPB) (Ajzen, 1991) describes situation specific mediators of behavior and posits that an individual's engagement is a function of attitudes (subjective evaluations on the behavior), subjective norms (perceived social appropriateness of the behavior) and perceived behavioral control (PBC) (ones' perceived ability to control the behavior) (Ajzen, 1991). It is attitudes, subjective norms and PBC that mediate between motivation and intention, where intention is the most proximal measurement of behavior and reflects the direction and intensity individuals plan to invest engaging in each behavior. There is a growing body of evidence (Chan et al., 2017) that supports the integrated SDT and TPB model showing subjects who reported higher autonomous regulation were more likely to endorse favorable attitudes and intentions to injury management behavior. The greater part of research in this area has investigated injury rehabilitation behavior and the influence of sports medicine practitioners, as significant others, on psychological need support and perceived autonomy support.

## Complex Systems Approach to Sports Injuries

More recently studies (Bittencourt et al., 2016; Stern et al., 2019) have called upon scientists to approach the inherent non-linearity of processes that are related to sports injuries and utilize approaches that integrate the complex systems approach into injury etiology research. A complex systems approach posits that sports injuries arise from the multifactorial complex interaction among a web of determinants and psychological behavior is conceptualized as a complex interplay between psychological and other factors. A complex systems approach to injury preventative behavior has the potential to identify profiles that characterize and constrain the interaction between SDT and TPB (Bittencourt et al., 2016). Investigations at the athlete level and coach level can provide insights that lead the improved understanding about the discrepancy between efficacy and effectiveness of injury prevention strategies (see Hulme and Finch, 2015). Applying a complex systems approach requires a coherent methodological approach that can account for the complex interaction between different variables (Bittencourt et al., 2016; Stern et al., 2019). This includes the statistical approach used to analyze the interaction between the variables studied. Machine learning has been suggested as an appropriate statistical approach (Bittencourt et al., 2016) because it does not necessarily yield one coefficient for a specific predictor but can show how a predictor can play a role in several different branches and has been used in previous injury-prevention research (Bittencourt et al., 2012). Neural networks are one form of machine learning that has gained a growing position in sports performance research (Lord et al., 2020) and has gained substantial footing in the field of psychological behavior (Epskamp et al., 2018). Neural networks have several advantages in comparison to more traditional statistical approaches such as multiple regression, logistic regression, and structural equation modeling (Chiang et al., 2006). One main advantage is that a both linear and non-linear relationships can be observed in neural networks. Also, neural networks do not require a priori assumptions about the relationships between independent and dependent variables (Xu et al., 2019). That is, the interpretation of results can be integrated with theoretical knowledge in the evaluation process but in a less formal way than other methods such as structural equation modeling (Shmueli and Koppius, 2011).

This complex systems approach could aid informing coaching practice through the development of intervention strategies to develop coaching environments that foster athletes' beliefs with respect to injury prevention, and, in turn, influence behavioral adherence. Creating coaching strategies that support athletes' autonomous regulation and/or enhance planned behavior to nurture sustainable self-regulated injury preventative behavior could be part of intervention strategies applied by golf coaches with golfers who have busy travel schedules and often play with pain.

## Aim of Study

The aim of the study was to investigate the potential interaction between perceived autonomy support, self-determined motivation, planned behavior, and how this relates to golfers self-reported intention of injury preventative behavior.

## Methods

### Participants

The 60 participants included in the study were on average 20.6 ± 5.1 years old and 24 of the 60 participants were women. The golfers were recruited by first taking contact with local coaches and then planning an information meeting between athletes, coaches, and a researcher (JP). During this information meeting athletes were informed verbally and given written information about the study and were invited to participate in the study.

### Inclusion Criteria

The inclusion criteria used in the selection of the participants were: (a) to be actively competing at a national level (senior players competing on the Swedish Golf Tour and junior players competing on the Teen Tour Elite competition series) or higher; and (b) to currently be in their late off-season or early pre-season.

### Data Collection Procedure

Firstly, local coaches were contacted and asked if they were interested in participation. After this, an information meeting between athletes, coach, and a researcher (JP) was planned. During this information meeting, athletes were informed verbally and given written information about the study and introduced to the App (Briteback, 2015). Thereafter, a paper was circulated around, and athletes were asked to write down their e-mail address if they were interested in participation. Participants answered the questionnaire through an APP (Briteback, 2015). The first questionnaire was then sent out at least 24 h this occasion. The first question of the first questionnaire included brief information about the study, ethical consideration, and participants were asked if they would like to continue.

### Questionnaires

The *background questionnaire* consisted of seven items and collected data on age, sex, golf experience, training time, handicap, level of competition, and injury history (Table 1).

**Table 1:** Background questions.

**Question**	**Answer selection provided**
How old are you?	NA
Select you sex	Man/Women
How long, in years, have you been playing golf?	NA
What is your current golf handicap?	NA
How many hours a week do you spend training for golf?	NA
Have you been injured during your sports career?	Never/Previously/Currently injured
What level are you currently playing golf at?	Regional (Participate in regional level competitions) National (participate in national competitions, e.g., national championships) International (participate in international competitions) World class (Ranked within the top 50 in the world)

The *Health Care Climate* (Williams et al., 1996) was used to collect data on perceived autonomy support. The questionnaire was in Swedish, the native language for all the participants. Items and instructions were translated from their original English version using the back-translation procedures described by Hambleton (2005). The McDonald's ω to measure internal consistency were calculated on the present sample. The questionnaire continued 6 items (e.g., my coach tries to understand me before he/she suggests any new ways to do things, I feel that my coach has given me choices and suggestions) (McDonald's ω = 0.911). All items were measured on a 7-point Likert scale ranging between 0 (*not true for me*) and 6 (*very true for me*).

The *Treatment Self-Regulation Questionnaire for Sports Injury Prevention* (Chan D. K. and Hagger, 2012) was used to collect data on autonomous motivation. The questionnaire was in Swedish, the native language for all the participants. Items and instructions were translated from their original English version using the back-translation procedures described by Hambleton (2005). The McDonald's ω to measure internal consistency were calculated on the present sample. The questionnaire measured two motivational regulations: controlled (six items, McDonald's ω = 0.843), and autonomous (five items, McDonald's ω = 0.728). All items were measured on a 7-point Likert scale ranging between 0 (*not true for me*) and 6 (*very true for me*).

Intention of the *Sport Injury Preventative Behavior questionnaire* (Chan D. K. C. and Hagger, 2012) was used to collect data on intentions of sports injury preventative behavior. The McDonald's ω to measure internal consistency were calculated on the present sample. The Intention questionnaire measured three components: frequency (five items, McDonald's ω = 0.624), effort (three items, McDonald's ω =0.660), and intention (three items, McDonald's ω =0.930), all items were measured on a 7-point Likert scale ranging between 0 (*not true for me*) and 6 (*very true for me*). A detailed explanation of the subset of questions used to measure injury preventative behavior is shown in Table 2.

**Table 2:** Questions used for the three components of injury preventative behavior.

**Frequency**	**Effort**	**Intention**
The following questions are related to how often you intend to work on preventing injuries during the coming 6 weeks	The following questions are related to how much effort you put into preventing injuries	The following questions are related to your plans to prevent injuries
How often do you intend to actively work with safety before training (e.g., checking equipment, checking the playing surface, using safety equipment)?	How much will you strive to train and play in safe sport environment (e.g., check equipment, checking the playing surface, using safety equipment)?	I will implement all the recommended procedures to reduce the risk of a sports injury.
How often do you intend to work on your physical and/or mental ability to avoid sports injuries (e.g., warm-up, strength training, getting enough rest, mental skills training)?	How much will you strive to improve your physical/mental fitness to avoid sports injuries (e.g., warming, stretching, physical exercise, resting enough)?	I will put a lot of focus on following the recommended procedures to reduce the risk of injury.
How often do you intend to work on not aggravating old injuries (e.g., ice, tape, rehab)?	How much will you strive to not aggravating old injuries (e.g., ice, tape, rehab)?	I plan to comply with all recommended procedures in the next 6 weeks to reduce the risk of sports injury.
How often do you intend to follow safety rules and regulations?		
How often do you intend to actively seek advice on injury prevention training from others (e.g., athletes, coaches, medical staff)?		

### Ethics

The study was approved by the Swedish Ethical Review Authority (Dnr:2019-02798). All participants received both verbal and written information before completing the consent forms and were informed of the possibility and right to terminate their participation at any time. All the participants gave written consent to participate in the study.

### Procedure

The participants answered all the questionnaires. The questionnaires were disseminated during February, which is during the off-season or early pre-season for most Scandinavian golfers.

### Statistical Analysis

The aim was to examine the interaction between the intended engagement in injury preventative behavior, perceived autonomy support, and two constructs of motivation. A neural network model analysis, using JASP (computer software JASP, Version 0.8.0.0), was performed to investigate the strength of connection between covariates (autonomy support, autonomous regulation, controlled regulation, effort of injury preventative behavior, frequency of injury preventative behavior, and intention of injury preventative behavior) and construct a network structure. Analysis of results was be performed by assessing edge strengths and node centrality to guide inference of the network topology. The sign of the edge weight (positive or negative) indicates the type of interaction, and the absolute value of the edge weight indicates the strength of the effect. The importance of individual nodes in the network can be assessed by node strength, closeness and betweenness along with visual interpretation of the network graph. Due to the relatively small sample size, and to control a Type I error, our study used a Gaussian graphical model (GGM) with a least absolute shrinkage and selection operator' (LASSO) (glasso; Friedman et al., 2008) following guidelines on estimating psychological networks suggested by Epskamp et al. (2018). Results from the network analysis are visualized using an *R-package* qgraph (Epskamp et al., 2012) in JASP. The position of the nodes in the network is based on the Fruchterman–Reingold algorithm (Fruchterman and Reingold, 1991) and inference methods from graph theory are used to assess which nodes are the most important in the network.

## Results

### Demographics

The participants had a handicap of 0.01 ± 2.6 strokes under par, 11.9 ± 5.7 years' playing competitive golf, spent 23.3 ± 9.4 h a week training for golf, and reported playing at national (*n* = 33), international (*n* = 26), and world-class level (*n* = 1). Of the 60 participants, eight were currently injured, 34 reported having a previous injury, and 18 had never been injured. Table 3 shows the mean scores for autonomy support, autonomous regulation, controlled regulation, effort, frequency, intention.

**Table 3:** Mean average scores for all psychological scales.

	**Autonomy support**	**Controlled regulation**	**Autonomous regulation**	**Intention**	**Frequency**	**Effort**
Mean	36.3 ± 6.0	18.2 ± 7.3	31.8 ± 3.4	8.1 ± 4.4	20.4 ± 4.9	13.4 ± 3.9

### Network Analysis

**Figure 1:**
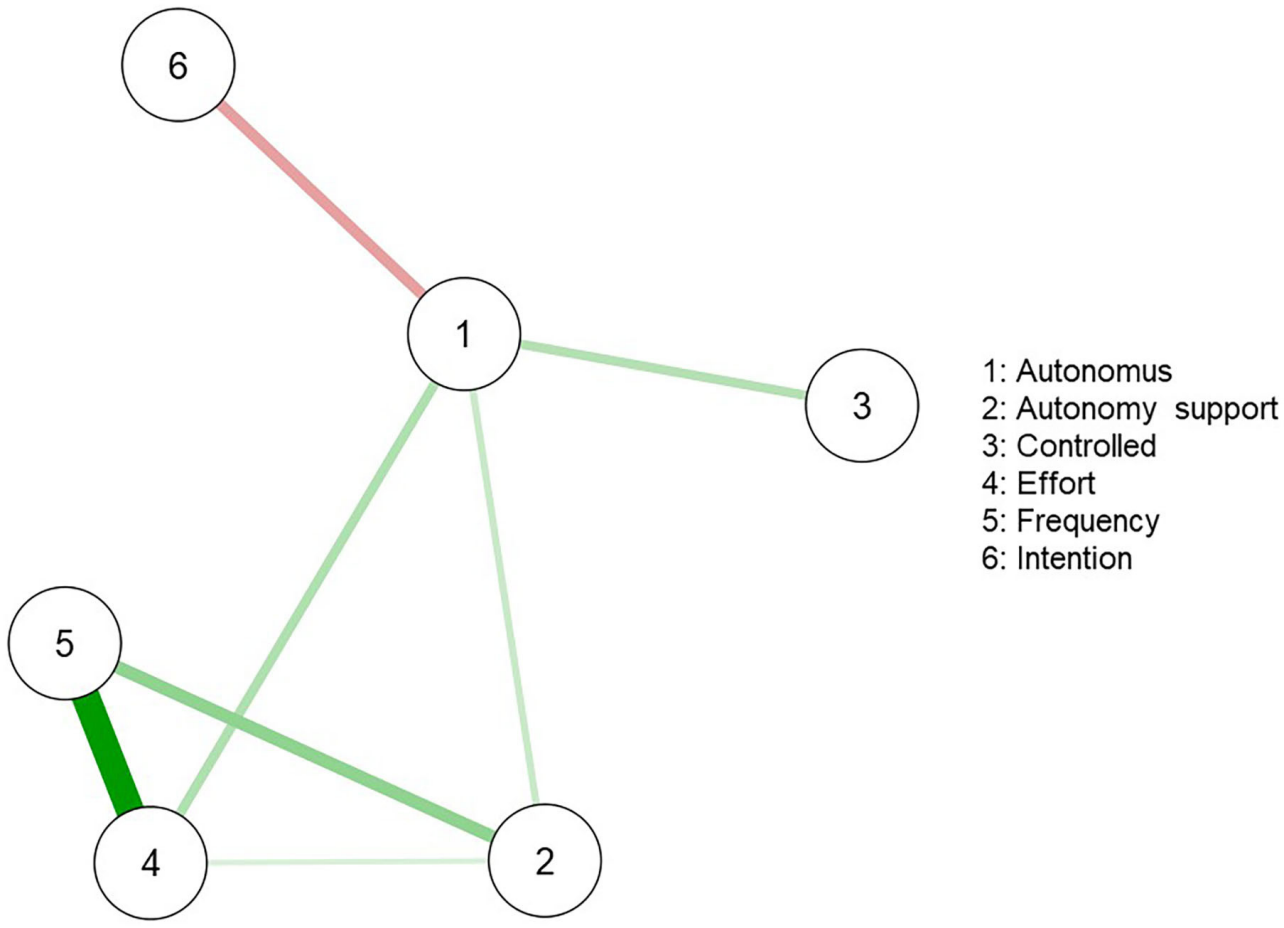
Motivation and injury preventative behavior network. Each node represents an item on the *injury preventative behavior questionnaire* (Chan et al., 2015), and each link represents the zero-order correlation between each pair of items. The thickness of a link represents the magnitude of the correlation and the colors represent the type of interaction (green = positive, red = negative). Autonomy support = 1, Autonomous regulation = 2, Controlled regulation = 3, Effort = 4, Frequency = 5, Intention = 6.

**Figure 2:**
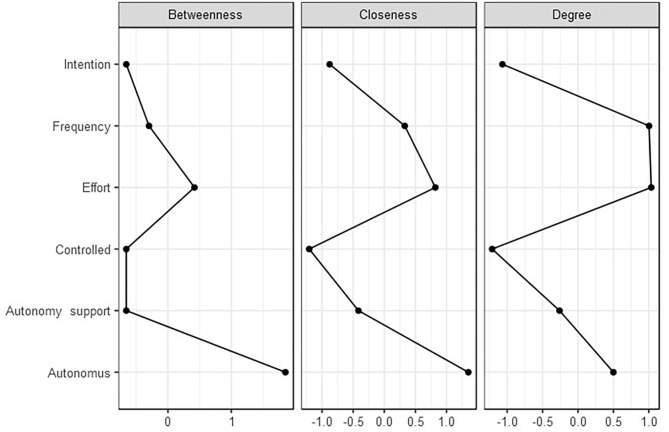
Centrality measures for *injury preventative behavior questionnaire* representing the betweenness, closeness, and strength of each node. Centrality indices are shown as standardized z-scores.

The network produced six nodes, Table 4 shows the weighted strength of interactions between all six nodes, eight of the 30 edges between the six nodes were estimated to be above zero. The strongest interaction was between the TPB constructs of effort and frequency (0.579). There were additional positive interactions between autonomy support and frequency (0.250), autonomous regulation and effort (0.177), autonomous regulation and controlled regulation (0.169), autonomous regulation and autonomy support (0.120), and autonomy support and effort (0.082). There was one negative interaction, and this was between autonomous regulation and intention (−0.212) Topological inference of the neural network revealed one possible cluster in the network (Figure 1). The cluster includes positive edges between perceived autonomy support, effort of injury preventative behavior, and frequency of injury preventative behavior. Autonomous regulation is situated in the center of the network (Figure 1) and exhibits the largest construct importance in the network (include betweenness score 1.8, Figure 2). In the first cluster the network revealed strong positive edges between frequency and effort of injury preventative behavior and between perceived autonomy support, frequency, and effort of injury preventative behavior (Figure 1).

**Table 4:** Edge-weights for the six nodes in the estimated network (Figure 1).

	**Autonomy support**	**Autonomous regulation**	**Controlled regulation**	**Effort**	**Frequency**	**Intention**
Autonomy support	0.000	0.120	0.000	0.082	0.250	0.000
Autonomous regulation	0.120	0.000	0.169	0.177	0.000	−0.212
Controlled regulation	0.000	0.169	0.000	0.000	0.000	0.000
Effort	0.082	0.177	0.000	0.000	0.579	0.000
Frequency	0.250	0.000	0.000	0.579	0.000	0.000
Intention	0.000	−0.212	0.000	0.000	0.000	0.000

## Discussion

We used a network analysis to investigate the interaction between perceived autonomy support, autonomous regulation, controlled regulation, and the frequency, effort, and intention of injury preventative behavior among high-level golfers. Our results indicate that autonomous motivation is the central node and exhibits the largest importance in the network. Our results also showed that perceived autonomy support is nested and positively associated with effort and frequency of injury preventative behavior whilst intention of injury preventative behavior is negatively associated with autonomous regulation.

Our main results show positive interactions between perceived autonomy support, effort of injury-preventative behavior, and frequency of injury-preventative behavior, that is, golfers who perceive greater autonomy support from their coaches show a greater propensity to undertake injury preventative behaviors. The fulfillment of psychological needs via perceived autonomy support is antecedent to autonomous regulation (i.e., feeling volitional and self-endorsed) and subsequently more persistent behavior (Deci and Ryan, 2002), in the case of our study more persistent injury preventative behaviors. The prospect of this persistent injury preventative behavior is illustrated in our findings via the cluster; perceived autonomy support, effort, and frequency. The strength of the edges and proximity between the constructs of effort and frequency illustrate how much the golfers are prepared to invest in engaging in injury preventative behavior. Together, this suggests that golfers who perceive greater autonomy support from their coach will aim to perform injury preventative behaviors more often than golfers who perceive lower levels of autonomy support from the coaches. Furthermore, golfers who reported having higher levels of autonomous regulation reported that they will try harder (effort) to perform injury preventative behaviors than golfers who reported lower levels of autonomous regulation. For coaches wanting to nurture autonomy, working to create an environment where the athletes can discuss both different types of stressors and other sport-related complaints (e.g., lack of recovery) will probably decrease the risk of injury (Ivarsson and Johnson, 2020).

The central node in the network is autonomous regulation and interacts with perceived autonomy support, controlled regulation, effort, frequency, and intention. The results from the network are, in general, aligned with the theoretical integration of SDT and TPB as proposed by Hagger and Chatzisarantis (2009). The results support evidence showing subjects with higher autonomous regulation are more likely to endorse favorable to injury management behavior (Chan et al., 2017). This model has also been demonstrated within sports injury research that has recognized athletes with greater autonomous regulation are more likely to undergo treatment because it is consistent with their internalized beliefs (Chan et al., 2011) and have an enhanced sense of happiness and excitement when returning to competition (Conti et al., 2019). Our investigation is, however, nuanced toward injury preventative behaviors and the influence of a significant other (coach) rather than injury treatment. This nuance may be important because coaches have an important nurturing sustainable injury preventative behavior for the golfers especially due to travel schedules that makes continued medical support problematic (O'Connor and Hawkes, 2013). Given the uncertainty surrounding all the stressors an athlete may experience and when these may lead to an injury, autonomous, self-regulated, and sustainable injury preventative behavior is a key factor for increasing the duration an athlete is free from injury.

Our secondary findings revealed a negative interaction between intention of injury preventative behavior and autonomous regulation. This negative interaction indicates golfers who report lower autonomous regulation report a greater intention to perform injury preventative behaviors which can be considered incongruent with SDT. This pattern of effects can potentially be explained by the TPB and it is plausible that a golfer's intention of injury preventative behavior is attributed to the relative influence of subjective norms and attitude, despite a lack of controlled regulation or autonomous regulation. Subjective norms are proposed to influence individual's behavior through a global perception of social pressure either to comply with the wishes of others or not (Ajzen, 1991). For instance, the perceived social pressure to perform injury preventative behaviors may have a positive influence on attitude and subsequently on intention, regardless of the motivational state of a golfer. Perceived risk and positive beliefs have also been shown to influence intention (Murphy et al., 2017) and the golfers in our study may relate positive beliefs and few negative outcomes with injury preventative behavior. Golfers who report high intention to injury preventative behavior and report lower autonomous regulation may be influenced by the relative influence of attitudes, subjective norms, and PBC. In this case, it is more appealing to adopt strategies to positively influence intention by create TPB influenced injury preventative behavior through enhancing attitudes, subjective norms, and PBC (Chan et al., 2017). An alternative interpretation of these results is related to the motivational training environment that golfers' practice in. Research has shown that the use of controlling behaviors by coaches is positively associated with athletes-controlled regulation and amotivation (Smith et al., 2016), and the social pressure moderating intention of injury preventative behavior may be controlling and lead to less persistent behaviors. Research in injury treatment behavior indicates that athletes with controlled reasons in sport were more likely to undertake injury treatment because they felt that the treatment was compulsory and must be done (Chan et al., 2011). These findings suggest that athletes can show intention to perform injury preventative behavior despite low autonomous regulation, however, this is judged to be a less sustainable behavior. These results need to be replicated in future studies before our results can be generalized to a larger population.

### Study Limitations

In the current study we applied complex system approach to motivation and injury prevention seems to result in promising data that nicely suits the interaction of SDT and TPB. One potential limitation with the analysis could be an increase in type II errors due to the relatively small sample size (*n* = 60) we had in the currents study. We accounted for this in the analysis by including the LASSO which uses regularizing penalty leading many edge estimates to shrink to zero and dropping out of the model. The final analyses return a sparse network with a relatively small number of edges and reduces the risk of overfitting the model (Epskamp et al., 2018). An additional potential limitation is the use of self-report to collect information about the injury preventive behaviors.

### Conclusions and Future Research

Practically, our results highlight the importance of coaches fostering perceived autonomy-supportive to promote injury preventative behavior among high-level golfers. Coaches' can nature this through; providing athletes with choice, giving opportunities for initiative-taking, giving a rationale for their actions, showing concern for the athlete both on and off the field (Banack et al., 2011). There were golfers who report lower autonomous regulation and high intention to injury preventative behavior. A strategy for coaches working with these golfers is to positively influence planned behavior through enhancing: (1) attitudes by promoting the advantages of injury preventative behavior; (2) social norms by appointing role models; and (3) PBC via improving the accessibility of injury prevention strategies through apps (Chan et al., 2017), for example, the FIFA 11+ (Sadigursky et al., 2017). We suggest that future research studies applying a complex systems approach to injury preventative behavior could use a longitudinal design and investigate the role of motivation and autonomy support on specific enacted behaviors within the context of injury history, particularly within sports where athletes seek pain relief for overuse injuries in order to tolerate continued play, such as golf. Furthermore, we recommend that future studies should consider controlled designs that independently manipulate each of the psychological constructs to provide evidence of the causal relationship within the integrated SDT TPB model and injury preventative behavior.

## Data Availability Statement

The raw data supporting the conclusions of this article will be made available by the authors, without undue reservation.

## Ethics Statement

The studies involving human participants were reviewed and approved by Regional Swedish Ethics Committee (Dnr:2019-02798). The patients/participants provided their written informed consent to participate in this study.

## Author Contributions

JP was responsible for data collection and data analysis. All authors have examined and agreed to the submitted version of the manuscript and contributed to study planning, interpretation of results, and drafting and finishing the manuscript.

## Conflict of Interest

The authors declare that the research was conducted in the absence of any commercial or financial relationships that could be construed as a potential conflict of interest.
